# Forschung an HNO-Universitätsklinika – Ergebnisse einer deutschlandweiten Umfrage zu Motivation, Hindernissen und Fördermöglichkeiten

**DOI:** 10.1007/s00106-025-01701-5

**Published:** 2026-01-12

**Authors:** C. H. L. Kürten, L. Boosfeld, L. Holtmann, S. Hansen, A. Daser, K. Stähr, S. Mattheis, T. Deitmer, T. Stöver, T. K. Hoffmann, Stephan Lang

**Affiliations:** 1 Universitätsklinikum Essen, Klinik für Hals-Nasen-Ohren-Heilkunde, Kopf- und Hals-Chirurgie, Universität Duisburg-Essen, Hufelandstraße 55, 45147 Essen, Deutschland; 2 HNOmedic PartG, Wittelsbacher Str. 2c, 82319 Starnberg, Deutschland; 3 Hals-Nasen-Ohrenpraxis am Waldweg, Waldweg 1, 37073 Göttingen, Deutschland; 4 Deutsche Gesellschaft für Hals-Nasen-Ohren-Heilkunde, Kopf- und Hals-Chirurgie e.V., Bonn, Deutschland; 5 Klinik für Hals‑, Nasen‑, Ohrenheilkunde, Universitätsmedizin Frankfurt, Goethe-Universität Frankfurt a. M., Frankfurt a. M, Deutschland; 6 Klinik für Hals‑, Nasen- und Ohrenheilkunde, Kopf- und Hals-Chirurgie, Universitätsklinik Ulm, Ulm, Deutschland

**Keywords:** Wissenschaftliche Kliniker, Klinische Wissenschaft, Forschungsförderung, Universitätsmedizin, Karriereentwicklung, Clinical scientist, Clinical research, Research support, Academic medical centers, Career development

## Abstract

**Hintergrund:**

Forschung ist ein zentraler Bestandteil der universitären Hals-Nasen-Ohren‑/HNO-Heilkunde. Gleichzeitig stehen klinisch tätige Ärztinnen und Ärzte unter zunehmendem Druck, wissenschaftliche Tätigkeiten mit steigenden Anforderungen im Klinikalltag zu vereinbaren.

**Ziel der Arbeit:**

Ziel der Studie war es, Motivation, Hemmnisse und strukturelle Rahmenbedingungen für Forschung und wissenschaftliche Qualifikation an deutschen HNO-Universitätsklinika zu analysieren und praxisnahe Empfehlungen zur Förderung abzuleiten.

**Material und Methoden:**

Eine anonymisierte Online-Umfrage wurde unter HNO-Ärztinnen und Ärzten an deutschen Universitätsklinika durchgeführt. Die Ergebnisse wurden deskriptiv ausgewertet.

**Ergebnisse:**

Von 131 Teilnehmenden arbeiteten 84 % in Vollzeit, 89 % waren promoviert, 28 % habilitiert. Während Promotionsinteressierte vor allem extrinsisch motiviert waren, überwogen bei Habilitationsinteressierten intrinsische Antriebe wie Erkenntnisinteresse und Freude an der Forschung. Haupthemmnisse für wissenschaftliche Tätigkeit waren Personalmangel, Dokumentationspflichten und fehlende geschützte Zeiträume. Forschung fand häufig außerhalb der regulären Arbeitszeit statt. Obwohl die wissenschaftliche Entwicklung regelmäßig durch die Leitungsebene thematisiert wurde, fühlten sich nur 36 % ausreichend unterstützt. 68 % sahen keinen Vorteil in der operativen Ausbildung durch wissenschaftliches Engagement.

**Schlussfolgerung:**

Die Ergebnisse verdeutlichen einen strukturellen Zielkonflikt zwischen klinischem Alltag und Forschung. Um HNO-Forschung an Universitätsklinika langfristig zu sichern, bedarf es gezielter Maßnahmen wie administrativer Entlastung, geschützten Forschungszeiten und transparenten Förderstrukturen.

## Abstract (en)

**Background:**

Research is a central pillar of academic otorhinolaryngology. However, clinicians increasingly face difficulties with integrating scientific activity into their demanding clinical routines.

**Objective:**

This study aimed to assess research motivation, perceived barriers, and structural conditions among clinicians at German university ENT departments and to derive practical recommendations for improvement.

**Materials and methods:**

An anonymized online survey was distributed among otorhinolaryngologists working in German academic hospitals. Data were analyzed descriptively.

**Results:**

Of 131 respondents, 84% worked fulltime, 89% held a doctoral degree, and 28% had completed habilitation. While extrinsic motivation dominated among doctoral candidates, habilitation aspirants were more driven by intrinsic factors such as scientific curiosity and enjoyment of research. Main obstacles included insufficient staffing, high documentation load, and lack of protected research time. Research was frequently conducted outside of regular working hours. Although research development was regularly addressed by management levels, only 36% felt adequately supported. Moreover, 68% of participants reported no perceived benefit of research engagement for their surgical training opportunities.

**Conclusion:**

These findings highlight a systemic tension between clinical demands and research. To ensure sustainable academic productivity in university ENT departments, institutional strategies such as protected time, reduction of the bureaucratic burden, and transparent support mechanisms are needed.

Forschung ist ein wesentlicher Bestandteil der universitären HNO-Heilkunde – steht im klinischen Alltag jedoch unter zunehmendem Druck. Diese Umfrage untersucht, welche Motivation Ärztinnen und Ärzte zu wissenschaftlicher Tätigkeit bewegt, welche strukturellen Hürden sie dabei erleben und wie gezielte Fördermaßnahmen die Forschungspraxis an Universitätsklinika verbessern könnten. Die Ergebnisse bieten praxisnahe Ansatzpunkte für Klinikleitungen, Fachgesellschaften und gesundheitspolitische Entscheidungsträger.

## Bedeutung von Forschung

Forschung und wissenschaftliches Arbeiten bilden einen zentralen Grundpfeiler der Hals-Nasen-Ohren-Heilkunde und sind essenziell für die kontinuierliche Weiterentwicklung des Fachs. Die Deutsche Forschungsgemeinschaft (DFG) definiert Wissenschaft als „systematische, nachvollziehbare und überprüfbare Suche nach neuen Erkenntnissen sowie deren Dokumentation und Veröffentlichung.“ In der medizinischen Forschung allgemein sowie in der Hals-Nasen-Ohren-Heilkunde besteht eine breite inhaltliche und methodische Vielfalt – von experimenteller Grundlagenforschung über klinische Studien und Versorgungsforschung bis hin zu epidemiologischen Analysen [21]. Dies trägt wesentlich zur Generierung evidenzbasierter Erkenntnisse und qualitativ hochwertigen Patientenversorgung bei.

Der Forschungsprozess selbst ist methodisch anspruchsvoll, verläuft selten linear und erfordert sorgfältige Planung, kritische Reflexion sowie interdisziplinäre Zusammenarbeit. Von der Entwicklung einer Fragestellung über das Studiendesign, die Datenerhebung und -auswertung bis hin zur Veröffentlichung und wissenschaftlichen Diskussion ist Forschung ein iterativer Prozess, der an Forschende hohe fachliche und organisatorische Anforderungen stellt [9]. Im klinischen Kontext, insbesondere bei patientennaher Forschung, ergeben sich zusätzliche Anforderungen – etwa durch ethische und regulatorische Rahmenbedingungen sowie die Integration in den laufenden Klinikbetrieb. Forschung findet zudem nicht isoliert statt, sondern ist eingebettet in ein komplexes institutionelles Gefüge aus Universitäten, Kliniken, Förderinstitutionen und berufsrechtlichen Vorgaben. Diese Strukturen bestimmen maßgeblich die Rahmenbedingungen wissenschaftlicher Arbeit, etwa durch Ressourcenverteilung, Anreizsysteme und infrastrukturelle Unterstützung [12]. Des Weiteren spielen akademische Qualifikationen, wie die Promotion und Habilitation, eine entscheidende Rolle für die Karriereentwicklung von Ärztinnen und Ärzten, insbesondere an universitären Einrichtungen [1, 37].

Im Idealfall ergeben sich Synergien zwischen Forschung, Patientenversorgung und Lehre – der Humboldt’schen Trias der universitären Medizin [38]. Sie bedingen und unterstützen sich gegenseitig und ermöglichen bestenfalls eine ganzheitliche, evidenzbasierte und innovative medizinische Praxis. In der klinischen Realität kommt es jedoch häufig zu Zielkonflikten zwischen diesen Bereichen. Zeitliche und finanzielle Ressourcen sind begrenzt, die Arbeitsverdichtung nimmt zu, und wissenschaftliche Tätigkeiten konkurrieren häufig mit den Anforderungen der Patientenversorgung und der Lehre [13]. Zu den strukturellen Belastungsfaktoren zählen unter anderem ein zunehmender administrativer Aufwand, wachsende regulatorische Anforderungen, ein hoher Bedarf an kontinuierlicher Weiterbildung sowie steigender ökonomischer Druck [25, 26]. Jüngere Erhebungen zur Berufszufriedenheit unter Medizinerinnen und Medizinern dokumentieren eine zunehmende Unzufriedenheit sowie eine Abkehr von der Tätigkeit in der Klinik oder von der Selbstständigkeit [39]. Spezifische Daten zur Situation klinisch tätiger Forschender in der HNO-Heilkunde fehlen jedoch bislang.

Vor diesem Hintergrund wurde eine Umfrage unter Ärztinnen und Ärzten an deutschen HNO-Universitätsklinika durchgeführt, um die Motivation zur wissenschaftlichen Qualifikation und Forschungstätigkeit zu analysieren, Barrieren sowie förderliche Faktoren im klinischen Forschungsalltag zu identifizieren und daraus Empfehlungen zur Verbesserung der Bedingungen für klinisch-wissenschaftlich tätige Ärztinnen und Ärzte abzuleiten.

## Material und Methoden

Zur Untersuchung der Motivation zur wissenschaftlichen Qualifikation, der Forschungsaktivität sowie der wahrgenommenen Förderfaktoren und Barrieren im klinischen Alltag wurde ein spezifischer Fragebogen entwickelt. Der Fragebogen wurde auf Basis der Erfahrungen des Autorenteams entwickelt und orientierte sich in Aufbau und Themenschwerpunkten an vergleichbaren Umfragen [1, 35, 37]. Die Auswahl der Fragen erfolgte konsensorientiert im Autorenteam. Eine formale Validierung wurde aufgrund des explorativen Studiendesigns nicht durchgeführt. Die Erhebung erfolgte als vollständig anonymisierte Online-Umfrage über die Plattform SurveyMonkey (FA. SurveyMonkey Inc., San Mateo/CA, USA).

Die Teilnehmenden wurden über den E‑Mail-Verteiler der Deutschen Gesellschaft für Hals-Nasen-Ohren-Heilkunde, Kopf- und Hals-Chirurgie (DGHNO) zur freiwilligen Teilnahme eingeladen. Eine explizite Begrenzung auf Mitglieder der DGHNO oder eine formale Verifikation der Universitätszugehörigkeit erfolgte nicht; durch die Formulierung der Einladung sowie die erhobenen Parameter (Karrierestufe, Promotions‑/Habilitationsstatus) war die Zielgruppe jedoch eindeutig adressiert. Die Befragung entsprach den Vorgaben der Datenschutz-Grundverordnung (DSGVO); die Studienleitung erhielt ausschließlich anonymisierte Datensätze.

Die erhobenen Daten wurden zur deskriptiven Auswertung in Microsoft Excel (Version 2010, Fa. Microsoft Corp., Redmond/WA, USA) überführt. Tabellen wurden mit Microsoft Excel bzw. Microsoft Word erstellt. Für manche Fragen wurde die Antwort in einer graduellen Antwortskala (Likert-Skala) von 1–5 erhoben; hier wird der gewichtete Mittelwert dargestellt.

Da keine personenbezogenen Daten erhoben, gespeichert oder analysiert wurden und eine Identifikation der Teilnehmenden ausgeschlossen war, wurde eine Beratung durch die zuständige Ethikkommission als nicht erforderlich eingestuft.

## Ergebnisse

### Teilnehmercharakteristika

Insgesamt wurden 131 vollständig ausgefüllte Fragebögen ausgewertet (Tab. 1). Der Anteil männlicher Teilnehmender betrug 50 % (*n* = 65), der weibliche Anteil 50 % (*n* = 66). 34 % (*n* = 44) befanden sich in Weiterbildung, 21 % (*n* = 27) waren Fachärztinnen und Fachärzte und 46 % (*n* = 60) oberärztlich tätig. 84 % (*n* = 110) arbeiteten in Vollzeit, 16 % (*n* = 21) in Teilzeit. Ein Großteil der Teilnehmenden war bereits promoviert (89 %, *n* = 117), die übrigen 11 % (*n* = 14) zeigten alle Interesse an einer Promotion. 28 % (*n* = 37) hatten bereits habilitiert; unter den nichthabilitierten Teilnehmenden äußerten 81 % (*n* = 76/94) Interesse an einer Habilitation.

**Tab. 1:** Teilnehmercharakteristika

Alle Personen, *n* = 131	
Geschlecht (m/w) *n* (%)	Männlich: *n* = 65 (50)
	Weiblich: *n* = 66 (50)
Medianes Alter in Jahren	36 (27–64)
Dienstgrad *n* (%)	Assistenzärzt:in: *n* = 44 (34)
	Fachärzt:in: *n* = 27 (21)
	Oberärzt:in: *n* = 60 (46)
Beschäftigungsgrad *n* (%)	Vollzeit: *n* = 110 (84)
	Teilzeit: *n* = 21 (16)
Akademischer Ausbildungsgrad *n* (%)	Nicht promoviert: *n* = 14 (11)
	Promoviert: *n* = 117 (89)
	Habilitiert: *n* = 37 (28)
Fach- und Oberärztinnen und Oberärzte, *n* = 90	
Stimmt das initiale Karriereziel noch mit dem aktuellen überein? *n* (%)	Ja: *n* = 57 (63)
	Nein: *n* = 33 (37)

### Forschungsmotivation und Qualifikationsziele

**Abb. 1:**
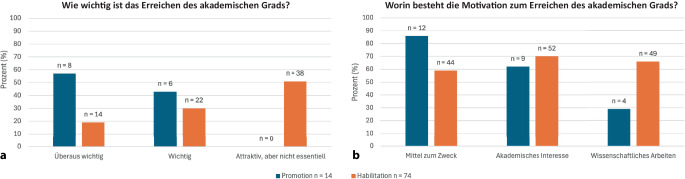
Motivation zum Erreichen eines akademischen Grads. Frage an nichtpromovierte (*n* = 14) und nichthabilitierte (*n* = 74) Studienteilnehmer zur Wichtigkeit (**a**) und zu den Motivationstreibern (**b**) zum Erreichen des jeweiligen akademischen Grads. Bei **b** Mehrfachantwort möglich

Die Promotion wurde insgesamt als bedeutsamer wahrgenommen als die Habilitation: Keiner der 14 nichtpromovierten Teilnehmenden hielt die Promotion für entbehrlich. Im Gegensatz dazu gaben 51 % (*n* = 38/74) der nichthabilitierten Befragten an, dass eine Habilitation nicht essenziell sei (Abb. 1). Die Motivation zur Promotion wurde überwiegend als Mittel zum Zweck angegeben (86 %, *n* = 12/14), bei der angestrebten Habilitation spielten hingegen akademisches Interesse (70 %, *n* = 52/74) und Freude am wissenschaftlichen Arbeiten (66 %, *n* = 49/74) eine größere Rolle (Abb. 1). Hauptgrund für die Tätigkeit an einer Universitätsklinik war bei vielen Befragten das operative Spektrum (Mittelwert: 4,2/5), gefolgt von Interesse an einer akademischen Laufbahn (3,7/5), Forschungsinteresse (3,6/5), Lehrtätigkeit (3,5/5) und dem Kollegenkreis (3,5/5; Tab. 2).

**Tab. 2:** Gründe für Tätigkeit an Uniklinik

	1	2	3	4	5	Gewichteter Mittelwert
	*n* (%)	*n* (%)	*n* (%)	*n* (%)	*n* (%)	
Interesse an der akademischen Laufbahn	15 (12)	12 (9)	21 (17)	34 (27)	45 (35)	*3,65*
Forschungsinteresse	14 (11)	14 (11)	25 (20)	29 (23)	45 (35)	*3,61*
Konservatives Spektrum	18 (14)	27 (21)	40 (32)	24 (19)	18 (14)	*2,98*
Operatives Spektrum	14 (11)	5 (4)	6 (5)	19 (15)	83 (65)	*4,20*
Lehrtätigkeit	10 (8)	20 (16)	27 (21)	36 (28)	34 (27)	*3,50*
Nettes Team	11 (9)	17 (13)	28 (22)	37 (29)	34 (27)	*3,52*
Work-Life-Balance	41 (32)	38 (30)	29 (23)	12 (9)	7 (6)	*2,26*
Standort	16 (13)	25 (20)	29 (23)	28 (22)	29 (23)	*3,23*
Bezug zur Klinik (z. B. PJ, Doktorarbeit)	47 (37)	12 (9)	16 (13)	26 (20)	26 (20)	*2,78*
Private Gründe	41 (32)	17 (13)	34 (27)	20 (16)	15 (12)	*2,61*

Antworten auf die Frage: „Warum haben Sie sich entschieden, an einer Uniklinik Ihre Ausbildung zu absolvieren? Bitte gewichten Sie […] mittels Werten von 1 (nicht wichtig) bis 5 (überaus wichtig)“; beantwortet: *n* = 127; übersprungen: *n* = 4

### Hemmende Faktoren im klinischen Forschungsalltag

**Abb. 2:**
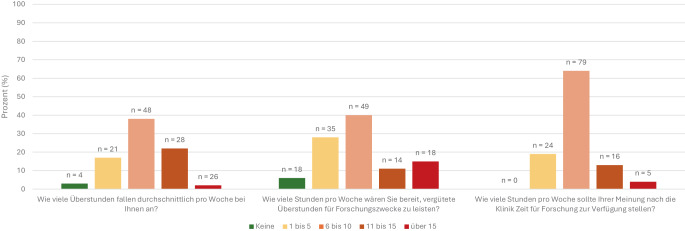
Überstunden und Forschungszeit. Fragen an die Umfrageteilnehmer zu im Wochenschnitt anfallenden Überstunden, zur Bereitschaft, vergütete Überstunden für Forschungszwecke zu leisten, und zur gewünschten wöchentlichen Freistellungsdauer für Forschungstätigkeiten

Als größte strukturelle Hemmnisse für wissenschaftliche Tätigkeit wurden eine hohe administrative Belastung (4,5/5) sowie unzureichende personelle Ressourcen (4,4/5) genannt (Tab. 3). Als weniger bedeutend wurden fehlende Förderung durch Vorgesetzte (3,1/5) oder eine mangelhafte Infrastruktur (2,5/5) bewertet. Bezüglich der Arbeitszeit gaben 22 % (*n* = 28/127) an, wöchentlich 11–15 Überstunden zu leisten, weitere 21 % (*n* = 26/127) sogar mehr als 15 h (Abb. 2). Weniger als eine Überstunde pro Tag machten nur 20 % (*n* = 26/127). Eine vollständige Vergütung dieser Überstunden erfolgte bei 27 % (*n* = 34), teilweise bei 45 % (*n* = 57) und gar nicht bei 25 % (*n* = 32).

**Tab. 3:** Forschungshemmende Faktoren

	1	2	3	4	5	Gewichteter Mittelwert
	*n* (%)	*n* (%)	*n* (%)	*n* (%)	*n* (%)	
Zu wenig Personal für den Klinikalltag	6 (5)	3 (2)	13 (11)	13 (11)	88 (72)	*4,41*
Zu viel Administration	4 (3)	6 (5)	2 (2)	27 (22)	84 (68)	*4,47*
Zu viel Lehrtätigkeit	29 (24)	30 (24)	45 (37)	14 (11)	5 (4)	*2,48*
Zu wenig fördernde Vorgesetzte	16 (13)	23 (19)	34 (28)	30 (24)	20 (16)	*3,12*
Zu wenig personelle Ressourcen (z. B. Study Nurses)	3 (2)	19 (15)	31 (25)	27 (22)	43 (35)	*3,72*
Fehlende Rückzugsmöglichkeiten für eigenes Arbeiten	24 (20)	31 (25)	16 (13)	19 (15)	33 (27)	*3,05*
Fehlende klinikinterne Infrastrukturen	36 (29)	36 (29)	20 (16)	12 (10)	19 (15)	*2,53*

Antworten auf die Frage: „Welche Faktoren halten Sie am häufigsten im klinischen Alltag von Forschungsarbeit ab? Bitte gewichten Sie […] mittels Werten von 1 (nicht relevant) bis 5 (überaus relevant).“; beantwortet: *n* = 123, übersprungen: *n* = 8

Als Hauptursachen für Überstunden wurden die umfangreiche Dokumentationspflicht (4,4/5), die Übernahme nichtärztlicher Tätigkeiten (3,7/5) sowie die Patientenversorgung (3,7/5) angegeben (Tab. 4). Forschungsbezogene Tätigkeiten wie Treffen mit Doktorandinnen und Doktoranden (2,9/5), klinische Studien (2,6/5) oder Labortätigkeiten (1,9/5) wurden deutlich seltener als Ursache genannt. Gleichzeitig zeigten sich viele Teilnehmende bereit, bei entsprechender Honorierung zusätzliche Zeit für Forschung aufzubringen: 65 % (*n* = 81/124) wären bereit, sechs oder mehr Stunden pro Woche aus ihrer Freizeit in wissenschaftliche Tätigkeit zu investieren, sofern diese als vergütete Überstunden angerechnet würden (Abb. 2).

**Tab. 4:** Gründe für Überstunden

	1	2	3	4	5	Gewichteter Mittelwert
	*n* (%)	*n* (%)	*n* (%)	*n* (%)	*n* (%)	
Patientenversorgung	8 (6)	9 (7)	36 (29)	30 (24)	42 (34)	*3,71*
Dokumentation	7 (6)	3 (2)	10 (8)	24 (19)	81 (65)	*4,35*
Nichtärztliche Tätigkeiten	10 (8)	21 (17)	13 (10)	28 (22)	53 (42)	*3,74*
Treffen mit Doktorandinnen und Doktoranden usw.	28 (22)	29 (23)	24 (19)	20 (16)	24 (19)	*2,86*
Klinische Studien	38 (30)	28 (22)	23 (18)	17 (14)	19 (15)	*2,61*
Labortätigkeit	78 (62)	14 (11)	14 (11)	8 (6)	11 (9)	*1,88*
Lehrtätigkeit	34 (27)	31 (25)	33 (26)	18 (14)	9 (7)	*2,50*
Fakultätsarbeit	75 (60)	20 (16)	10 (8)	10 (8)	10 (8)	*1,88*
Vollendung Promotion bzw. Habilitation	71 (57)	11 (9)	15 (12)	12 (10)	16 (13)	*2,13*

Antworten auf die Frage: „Wodurch kommen […] Überstunden zustande? Bitte gewichten Sie […] mittels Werten von 1 (nicht relevant) bis 5 (überaus relevant)“; Beantwortet: *n* = 125, Übersprungen: *n* = 6

### Fördermöglichkeiten und institutionelle Unterstützung

64 % (*n* = 79/124) der Befragten gaben an, dass ihre wissenschaftliche Entwicklung regelmäßig durch die Klinikleitung thematisiert werde, etwa im Rahmen von Mitarbeitergesprächen (Tab. 5). Dennoch fühlten sich nur 36 % (*n* = 45/124) insgesamt ausreichend in ihrer Forschungstätigkeit unterstützt. Die Möglichkeit zur Teilnahme an nationalen oder internationalen Kongressen wurde von 77 % (*n* = 95/124) regelmäßig wahrgenommen. Eine vollständige finanzielle Unterstützung erhielten jedoch nur 10 % (*n* = 12), 40 % (*n* = 50) erhielten eine anteilige Finanzierung, während 49 % (*n* = 61) keinerlei finanzielle Unterstützung erhielten (Tab. 5). Sonderurlaubstage wurden bei 31 % (*n* = 38) vollständig, bei 51 % (*n* = 63) teilweise und bei 15 % (*n* = 19) gar nicht gewährt (Tab. 5). 69 % (*n* = 86/124) empfanden die Förderpraxis innerhalb der Klinik als ungleich verteilt. Etwa ebenso viele (68 %, *n* = 84/124) gaben an, dass wissenschaftliche Aktivität nicht mit einer bevorzugten chirurgischen Ausbildung einhergehe (Tab. 5). Ein strukturiertes Förderprogramm im Sinne eines Clinician-Scientist-Tracks wurde von 79 % (*n* = 96/122) als attraktiv bewertet. An 83 % (*n* = 101/122) der Institutionen war ein solches Programm bereits etabliert.

**Tab. 5:** Fördermaßnahmen

		Ja		Nein		kA
		*n* (%)		*n* (%)		*n* (%)
Wird Ihre wissenschaftliche bzw. akademische Entwicklung regelmäßig besprochen? (*n* = 124)		79 (64)		40 (32)		5 (4)
Fühlen Sie sich durch Ihren Arbeitgeber in Bezug auf Forschung ausreichend unterstützt? (*n* = 124)		45 (36)		64 (52)		15 (12)
Werden Ihrer Meinung nach alle Teamkolleginnen und -kollegen gleichermaßen gefördert? (*n* = 124)		34 (27)		86 (69)		4 (3)
Werden wissenschaftlich arbeitende Assistenzärztinnen und -ärzte im klinischen Alltag/der operativen Ausbildung stärker gefördert? (*n* = 124)		31 (25)		84 (68)		9 (7)
Wäre ein klinisch-wissenschaftliches Förderprogramm für Sie interessant? (*n* = 122)		96 (79)		26 (21)		–
Existieren an Ihrer Universität Freistellungsangebote für Forschungsinitiativen, z. B. Clinician-Scientist-Programme? (*n* = 122)		101 (83)		21 (17)		–
Erhalten Sie regelmäßig die Möglichkeit, nationale oder internationale Kongresse/Kurse zu besuchen? (*n* = 124)		95 (77)		23 (19)		6 (5)
–	Vollständig		Teilweise	Nein	kA	
	*n* (%)		*n* (%)	*n* (%)	*n* (%)	
Erhalten Sie bei Teilnahme an nationalen oder internationalen Kongressen eine finanzielle Unterstützung? (*n* = 124)	12 (10)		50 (40)	61 (49)	1 (1)	
Erhalten Sie bei Teilnahme an nationalen oder internationalen Kongressen Sonderurlaubstage bzw. gelten die Tage als Arbeitstage für die Zeit des Kongresses? (*n* = 124)	38 (31)		63 (51)	19 (15)	4 (3)	

*kA* keine Angabe

## Diskussion

### Forschung im aktuellen ärztlichen universitären Arbeitsumfeld

Die Ergebnisse dieser Umfrage verdeutlichen einen strukturellen Zielkonflikt zwischen wissenschaftlicher Tätigkeit und den realen Arbeitsanforderungen im universitären Klinikalltag. Viele Teilnehmende berichteten von einer hohen Anzahl unbezahlter oder nur teilweise vergüteter Überstunden, die in erster Linie durch nichtwissenschaftliche Aufgaben wie Dokumentation, Verwaltung oder nichtärztliche Tätigkeiten verursacht werden.

Dieser Befund spiegelt sich in aktuellen Entwicklungen des Gesundheitssystems wider: Die zunehmende Ökonomisierung [25, 31], ein exponentiell wachsendes medizinisches Wissen [11], steigende Dokumentationspflichten [26], neue Technologien [4, 7] sowie Personalengpässe infolge von Teilzeitmodellen [30] oder unbesetzten Stellen belasten die klinische Arbeit zunehmend. Aktuelle Simulationen des Einflusses der Krankenhausreform auf die HNO-Kliniklandschaft lassen eine Verschärfung dieser Belastungsfaktoren erwarten [24]. Diese Faktoren wirken häufig kumulativ und verstärken sich wechselseitig [19], sodass für als weniger akut wahrgenommene, aber strategisch wichtige Tätigkeiten wie Forschung kaum noch zeitliche oder kognitive Freiräume bestehen.

Vor allem jüngere Ärztinnen und Ärzte erleben diese Verdichtung als erhebliche Belastung – unabhängig davon, ob sie aktiv wissenschaftlich tätig sind. Verschiedene Erhebungen zeigen eine zunehmende berufliche Unzufriedenheit sowie eine wachsende Bereitschaft, die ärztliche Tätigkeit – zumindest im stationären oder universitären Umfeld – zu verlassen [3, 6, 22].

In diesem Spannungsfeld aus wachsender Komplexität, knappen Ressourcen und steigenden Anforderungen gerät die wissenschaftliche Weiterentwicklung eines Fachs wie der Hals-Nasen-Ohren-Heilkunde somit in Gefahr, sofern keine gezielte strukturelle Unterstützung erfolgt [23]. Die vorliegende Umfrage zeigt jedoch auch: Das Interesse an wissenschaftlicher Tätigkeit ist grundsätzlich vorhanden – insbesondere bei jüngeren Kolleginnen und Kollegen

### Intrinsische versus extrinsische Forschungsmotivation

In der Motivationspsychologie wird zwischen intrinsischer Motivation – also Handeln aus Interesse, Neugier oder Freude am Erkenntnisgewinn – und extrinsischer Motivation, etwa durch Status, berufliche Vorteile oder äußeren Druck, unterschieden [10]. Beide Motivationsarten beeinflussen auch die Entscheidung zum wissenschaftlichen Arbeiten, jedoch in unterschiedlicher Gewichtung entlang des akademischen Karriereverlaufs. In der vorliegenden Umfrage bewerteten 86 % der noch nicht promovierten Ärztinnen und Ärzte den Doktortitel primär utilitaristisch („Mittel zum Zweck“). Bei den habilitationsinteressierten Teilnehmenden dominierten hingegen das akademische Erkenntnisinteresse (70 %) sowie die Freude am wissenschaftlichen Arbeiten (66 %) als Hauptmotive. Diese Verschiebung deutet darauf hin, dass intrinsische Beweggründe im Verlauf der akademischen Entwicklung an Bedeutung gewinnen.

Diese Beobachtung findet sich auch in anderen Erhebungen: In einer bayerischen Umfrage unter 201 medizinischen Promovierenden dominierten extrinsische Motive – etwa dass eine Promotion „im Fach üblich“ sei (88 %) oder „verbesserte berufliche Chancen“ bringe (86 %) [8]. Im Gegensatz dazu gaben in einer Befragung unter 707 Habilitierten in der Medizin 82 % die „Freude an der Forschung“, ein intrinsisch geprägtes Motiv, als primären Antrieb an [37]. Ob jedoch eine ursprünglich hohe intrinsische Motivation zur Habilitation führt oder ob sich intrinsische Motivation erst im Zuge zunehmender wissenschaftlicher Tätigkeit entwickelt, lässt sich anhand dieser Querschnittsdaten nicht eindeutig klären. Prospektive Studien bieten hierzu weiterführende Hinweise: In einer Untersuchung unter 315 Medizinstudierenden war nur die intrinsische Motivation – nicht aber die extrinsische – nach multivariater Regression signifikant mit einer erhöhten Forschungstätigkeit assoziiert (OR = 2,5; 95 %-KI: 1,35–4,78) [33]. Ergänzend zeigte eine kombinierte Befragung und Interviewstudie mit 579 Studierenden und 23 Einzelinterviews, dass forschungsaktive Studierende insbesondere durch die internalisierte Wahrnehmung von Kompetenz (z. B. Selbstwirksamkeit) und Verbundenheit (z. B. klinische Relevanz, soziale Bedeutung der Forschung) motiviert waren – beides zentrale Elemente intrinsischer Motivation [35].

Somit gilt für die Förderung wissenschaftlicher Tätigkeit in der HNO-Heilkunde: Extrinsische Anreize – etwa akademische Titel oder äußerer Druck – können initial wirksam sein, entscheidend für eine nachhaltige Forschungstätigkeit ist jedoch die Verankerung intrinsischer Motivatoren. Diese lassen sich stärken durch die Wertschätzung wissenschaftlicher Leistungen, das Erleben fachlicher Selbstwirksamkeit, die Verknüpfung von Forschung mit klinischer Relevanz sowie ein unterstützendes Arbeitsumfeld, welches hierfür die organisatorischen Rahmenbedingungen schafft.

### Forschung als Opfer ineffizienter klinischer Strukturen

Die vorliegenden Umfrageergebnisse deuten darauf hin, dass wissenschaftliches Arbeiten im Klinikalltag weniger durch andere ärztliche Kernaufgaben in Lehre und Krankenversorgung erschwert wird, sondern in erster Linie durch organisatorische Rahmenbedingungen. Die berichtete hohe Zahl an (oftmals unbezahlten) Überstunden wurde überwiegend auf administrative Aufgaben, insbesondere Dokumentationspflichten sowie die Übernahme nichtärztlicher Tätigkeiten, zurückgeführt. Die Zuordnung als „nichtärztliche Tätigkeit“ erfolgte auf Basis der subjektiven Einschätzung der Befragten und wurde nicht von der Umfrage definiert. Forschungsbezogene Tätigkeiten spielten hingegen eine nachgeordnete Rolle bei der Entstehung dieser Mehrbelastung. Zudem wäre eine Mehrzahl der Befragten bereit, Forschung außerhalb der regulären Arbeitszeit zu leisten, sofern dies vergütet wird. Gleichwohl ist eine solche Nutzung der Freizeit rechtlich und strukturell problematisch: Eine tarifkonforme, vergütete Forschungsleistung außerhalb der Regelarbeitszeit ist nicht vorgesehen. Vielmehr sollten Universitätsklinika vorhandene strukturelle Mittel – wie den Zuführungsbetrag der Länder zur Förderung von Forschung und Lehre – nutzen, um geschützte Forschungsanteile innerhalb der regulären Arbeitszeit zu schaffen. Die Ergebnisse unserer Studie sprechen somit weniger für ein Vergütungs-, als vielmehr für ein Verteilungsproblem vorhandener Ressourcen.

Diese Einschätzung deckt sich mit empirischen Studien zur Arbeitszeitverwendung von Ärztinnen und Ärzten im Klinikalltag. In einer Beobachtungsstudie an einem deutschen Krankenhaus wurden 13 Ärztinnen und Ärzte über circa 37 h begleitet. Die Auswertung ergab, dass 37 % der Arbeitszeit mit der elektronischen Patientenakte verbracht wurden, aber nur 28 % im direkten Patientenkontakt [17]. Eine weitere Beobachtung von neun Ärztinnen und Ärzten aus drei Fachrichtungen zeigte ebenfalls, dass diese im Durchschnitt 93,1 min täglich (19 %) für Dokumentation aufwendeten, aber lediglich 33,8 min (7 %) für direkten Patientenkontakt [36].

Zahlreiche Interventionsstudien belegen, dass gezielte organisatorische Maßnahmen zu erheblicher Entlastung führen können. So konnte durch die Einführung eines IT-gestützten Tools zur automatisierten Erstellung von Stationslisten die Erstellungszeit von vormals 121 min auf rund 5 min verkürzt werden [20]. Ebenso effektiv zeigte sich die Delegation dokumentationsbezogener Aufgaben: In einer 12-monatigen Crossover-Studie war der Einsatz medizinischer Schreibkräfte mit deutlich weniger Dokumentation außerhalb der regulären Arbeitszeit assoziiert. Zudem war die Wahrscheinlichkeit, dass ein Arzt seine Dokumentation rechtzeitig abschloss, signifikant erhöht (OR = 2,8; 95 %-KI: 1,2–7,1) [28, 29].

Neben spezifischen Maßnahmen wie Digitalisierung oder Delegation lassen sich auch durch ganzheitliche Prozessoptimierung relevante Effekte erzielen. Das so genannte Lean-Hospital-Konzept, eine Übertragung des Lean-Managements auf den klinischen Kontext, zielt darauf ab, Prozesse zu verschlanken, unnötige Schritte zu eliminieren und Aufgaben gezielt auf unterschiedliche Berufsgruppen zu verteilen [34]. Lean-Hospital-Interventionen werden in wissenschaftlichen Untersuchungen überwiegend als positiv bewertet [27]. Eine US-amerikanische Längsschnittstudie zeigte, dass die Bildschirmzeit von Hausärzten nach Einführung von Lean-Methoden um 10 % im ersten Jahr und die Gesamtarbeitszeit um 20 % nach drei Jahren sanken [18]. Auch im HNO-spezifischen Kontext gibt es vielversprechende Ansätze: Die Umstrukturierung einer onkologischen Kopf-Hals-Ambulanz von einer sequenziellen zu einer interdisziplinär organisierten Versorgung führte unter Anwendung von Lean-Prinzipien zu einer signifikanten Verkürzung der Zeitspanne zwischen Erstvorstellung und Therapiebeginn [15].

Die Einschätzung der Umfrageteilnehmenden, dass ineffizient gestaltete Arbeitsabläufe, hohe Dokumentationslast und mangelnde Delegation ärztliche Zeitressourcen erheblich binden – zulasten sowohl der Patientenversorgung als auch der Forschung –, wird durch diese Studien gestützt. Zur Förderung von Forschung gehört somit auch, klinisch-organisatorische Prozesse zu evaluieren und gezielt so umzugestalten, dass genuine ärztliche und wissenschaftliche Aufgaben wieder in den Vordergrund rücken.

### Wissenschaft und operative Ausbildung – Zielkonflikt oder Synergiepotenzial?

Im universitären Kontext stellt die chirurgische Tätigkeit eine zentrale ärztliche Aufgabe in der HNO-Heilkunde dar – sowohl hinsichtlich des zeitlichen Umfangs als auch des mit ihr verbundenen Ausbildungs- und Ressourcenaufwands. Eine fachärztliche Qualifikation erfolgt nicht allein über theoretisches Wissen oder diagnostische Fähigkeiten, sondern wesentlich über die schrittweise Aneignung praktisch-chirurgischer Kompetenzen. Diese sind – anders als beispielsweise das Erlernen von Faktenwissen – stark abhängig von externen Rahmenbedingungen: feste Zeitfenster im Operationssaal, spezifische Fallzahlen sowie qualifizierte Anleitung und Supervision [5]. Die Ergebnisse der vorliegenden Umfrage zeigen, dass viele Teilnehmende das operative Spektrum als Hauptgrund für ihre Tätigkeit an einer Universitätsklinik nannten. Dennoch empfanden nur 36 % die Förderung durch die Klinikleitung als ausreichend, und 68 % sahen keinen Vorteil in der operativen Ausbildung durch wissenschaftliches Engagement.

Diese Diskrepanz weist auf ein grundlegendes Problem hin: Wissenschaftlich tätige Ärztinnen und Ärzte investieren Zeit außerhalb des klinischen Betriebs, was zu zeitweiliger Abwesenheit vom OP und damit zu einem gefühlten oder realen Verlust an operativer Routine („skill decay“) führen kann [2]. Diese Wahrnehmung wird durch Umfragen in anderen chirurgischen Fächern gestützt: Insbesondere bei längeren Forschungsphasen berichten Weiterbildungsassistentinnen und -assistenten regelmäßig über einen Kompetenzverlust im operativen Bereich sowie Unsicherheiten beim klinischen Wiedereinstieg [14]. Zugleich eröffnet dieses Spannungsfeld auch Gestaltungsspielräume: Wenn wissenschaftliches Engagement gezielt durch operative Förderung honoriert wird, kann daraus ein förderlicher Anreizmechanismus entstehen. Einige Weiterbildungsstätten haben bereits reagiert und bieten „chirurgische Rehabilitationsmodule“ an [32] – beispielsweise durch Simulationstrainings nach Forschungsphasen [16]. Solche Maßnahmen können den Wiedereinstieg in den klinisch-operativen Alltag erleichtern und verhindern, dass forschungsaktive Nachwuchschirurginnen und -chirurgen langfristig ins Hintertreffen geraten. Gleichzeitig kann durch eine gezielte Förderung von forschenden Ärztinnen und Ärzten auch die Motivation für wissenschaftliches Arbeiten erhalten werden.

Letztlich wird von forschenden Chirurginnen und Chirurgen erwartet, eine doppelte Expertise aufzubauen: operative Fähigkeiten und wissenschaftliche Kompetenz. Dieses hohe Anspruchsprofil ist nur erfüllbar, wenn es institutionell gefördert, durch Anreize gestützt und organisatorisch ermöglicht wird. Ein mögliches Mittel, die Bewerbung um und Teilnahme an Clinician-Scientist-Programmen, wurde von 101 (83 %) der Teilnehmenden als an ihrer Institution vorhanden angegeben.

### Limitationen der Studie

Diese Untersuchung unterliegt mehreren Einschränkungen, die bei der Interpretation der Ergebnisse berücksichtigt werden sollten. Erstens handelt es sich um eine querschnittliche Erhebung auf Basis einer freiwilligen Online-Umfrage. Daraus ergibt sich eine potenzielle Selbstselektion der Teilnehmenden, mit möglicherweise über- oder unterdurchschnittlichem Interesse an wissenschaftlichen Fragestellungen. Da zudem der genaue Umfang des angeschriebenen E‑Mail-Verteilers nicht bekannt ist und keine Rücklaufquote berechnet werden konnte, lässt sich keine statistische Repräsentativität ableiten.

Zweitens erfolgte keine systematische Erfassung regionaler oder struktureller Unterschiede zwischen den verschiedenen teilnehmenden Kliniken. Insbesondere Faktoren wie Klinikgröße, akademisches Profil, chirurgische Schwerpunkte, Drittmittelausstattung oder spezifische Förderungskonzepte wurden nicht differenziert erhoben. Zudem adressiert die Umfrage nicht eine mögliche kritische Haltung gegenüber bestehenden Forschungsstrukturen und wissenschaftlicher Praxis, wie die häufig kritisierte Fokussierung auf Publikationsquantität statt -qualität, die ungleiche Anerkennung von Studienleistungen, die Schwerpunktsetzungen durch Drittmittellogik oder die als ungerecht wahrgenommene Prestige bestimmter Themen und Methoden. Solche kritischen Wahrnehmungen oder Vorurteile könnten selbst eine relevante Barriere für wissenschaftliches Engagement darstellen.

Drittens war keine Gegenprüfung anhand objektiver Daten (z. B. Publikationszahlen, Drittmitteleinwerbung, Dienstplänen, Op.-Katalog) möglich. Eine Verknüpfung der subjektiven Einschätzungen der Teilnehmenden mit quantitativen Leistungskennzahlen hätte potenziell Rückschlüsse auf die tatsächliche Produktivität innerhalb der jeweiligen Kliniken ermöglicht. Künftige Untersuchungen sollten daher Selbstauskünfte mit leistungsbezogenen Daten kombinieren, um Korrelationen zwischen wahrgenommener Förderung und tatsächlicher Forschungsaktivität zu ermöglichen.

Trotz dieser Limitationen liefert die Umfrage wertvolle Einsichten in die aktuelle Wahrnehmung wissenschaftlicher Tätigkeit an deutschen HNO-Universitätsklinika und bildet eine Grundlage für zukünftige, auch interventionelle, Studien.

## Ausblick

Die Ergebnisse dieser Umfrage unterstreichen den Bedarf nach strukturellen Anpassungen, um wissenschaftliche Tätigkeit im klinischen Alltag langfristig zu ermöglichen. Künftige Studien sollten untersuchen, welche spezifischen Maßnahmen – etwa geschützte Forschungszeit, strukturierte Mentoringprogramme oder gezielte operative Förderung forschungsaktiver Ärztinnen und Ärzte – wirksam zur Steigerung der wissenschaftlichen Produktivität beitragen. Eine Evaluation solcher Förderstrukturen ist relevant, um akademische Karrieren im Spannungsfeld von Klinik, Lehre und Forschung nachhaltig zu unterstützen.

## Fazit für die Praxis

- Die Motivation zur wissenschaftlichen Arbeit unter HNO-Ärztinnen und Ärzten an Universitätsklinika ist hoch.
- Hauptbarrieren sind Personalmangel, Belastung durch nichtärztliche Tätigkeiten und fehlende zeitliche Freiräume.
- Forschung wird oft außerhalb der regulären Arbeitszeit betrieben, aber viele wären bereit, Freizeit für vergütete Forschungszeit zu investieren.
- Der Wunsch nach strukturierten Förderformaten ist weit verbreitet.
- Klinikleitungen/Fakultäten sollten geschützte Forschungszeiten einführen, administrative Aufgaben delegieren und individuelle Forschungsziele regelmäßig thematisieren.
- Transparente und gerechte Förderstrukturen innerhalb von Teams könnten Motivation, Karriereaussichten und die wissenschaftliche Produktivität stärken.

## Data Availability

Die Rohdaten, auf denen die Schlussfolgerungen dieses Artikels basieren, werden von den Autoren ohne unangemessene Vorbehalte, auf Nachfrage, zur Verfügung gestellt.
